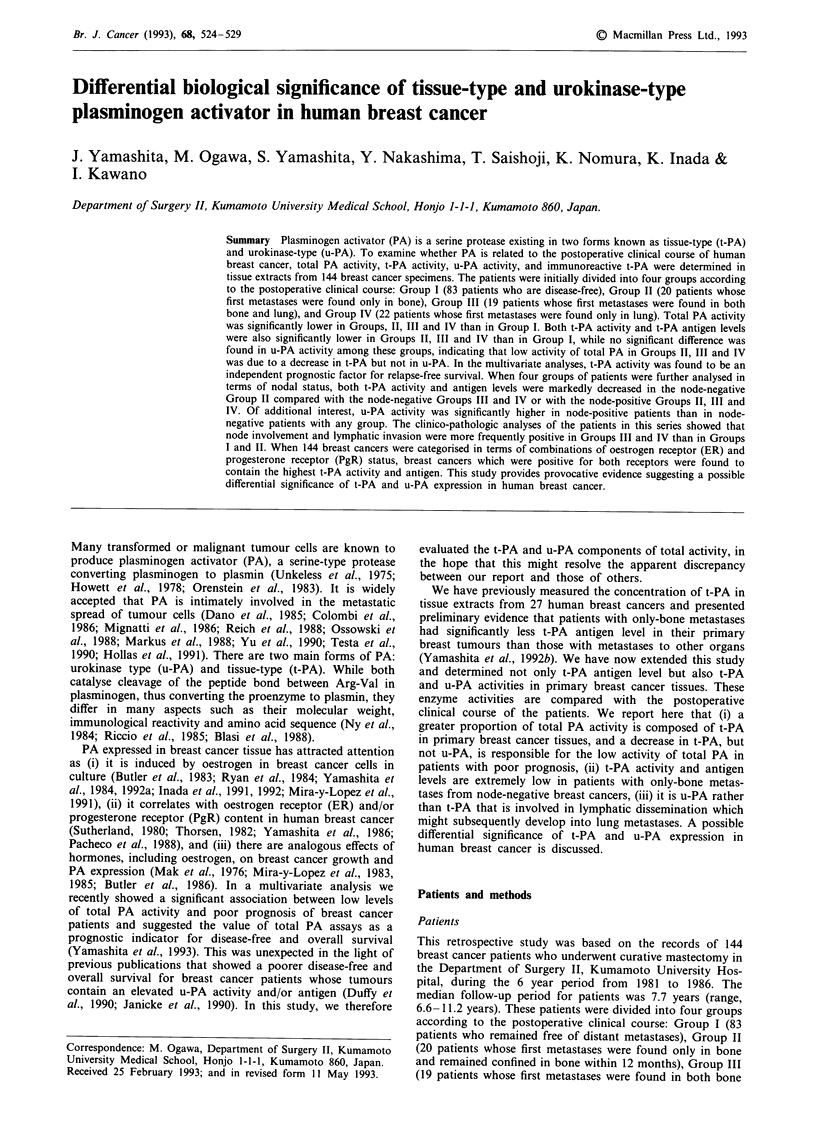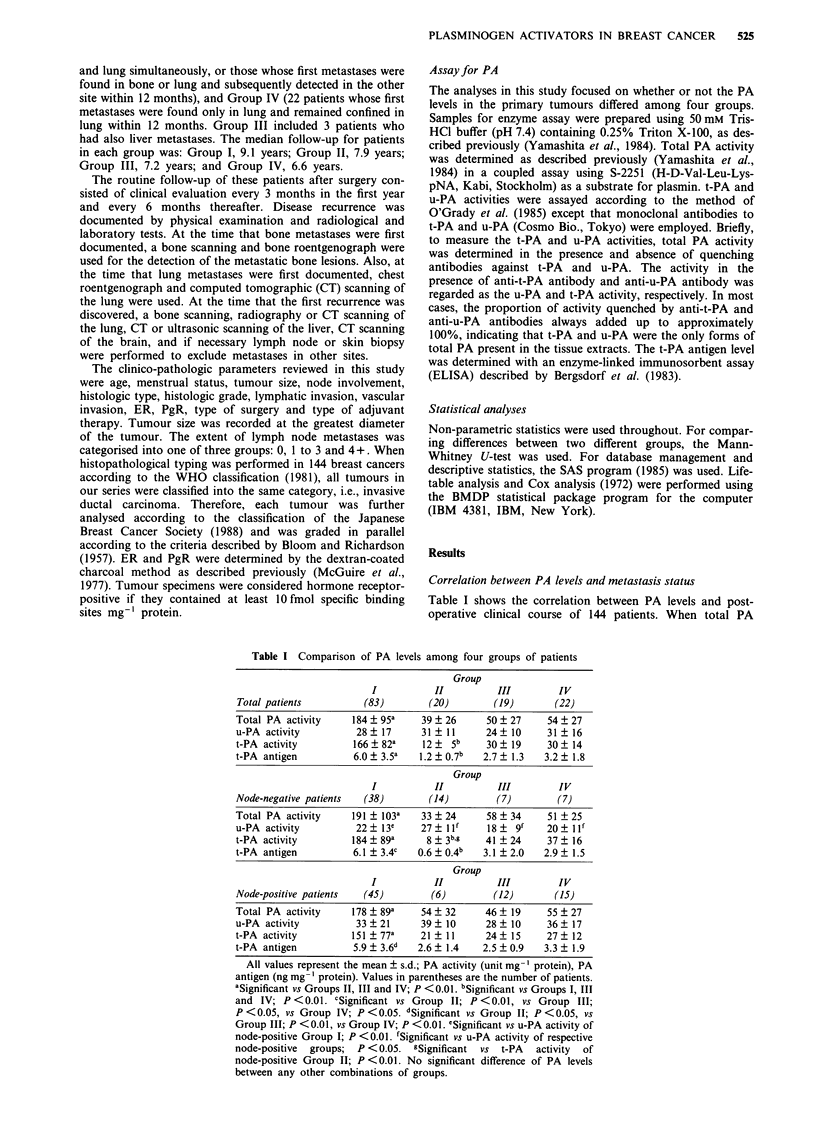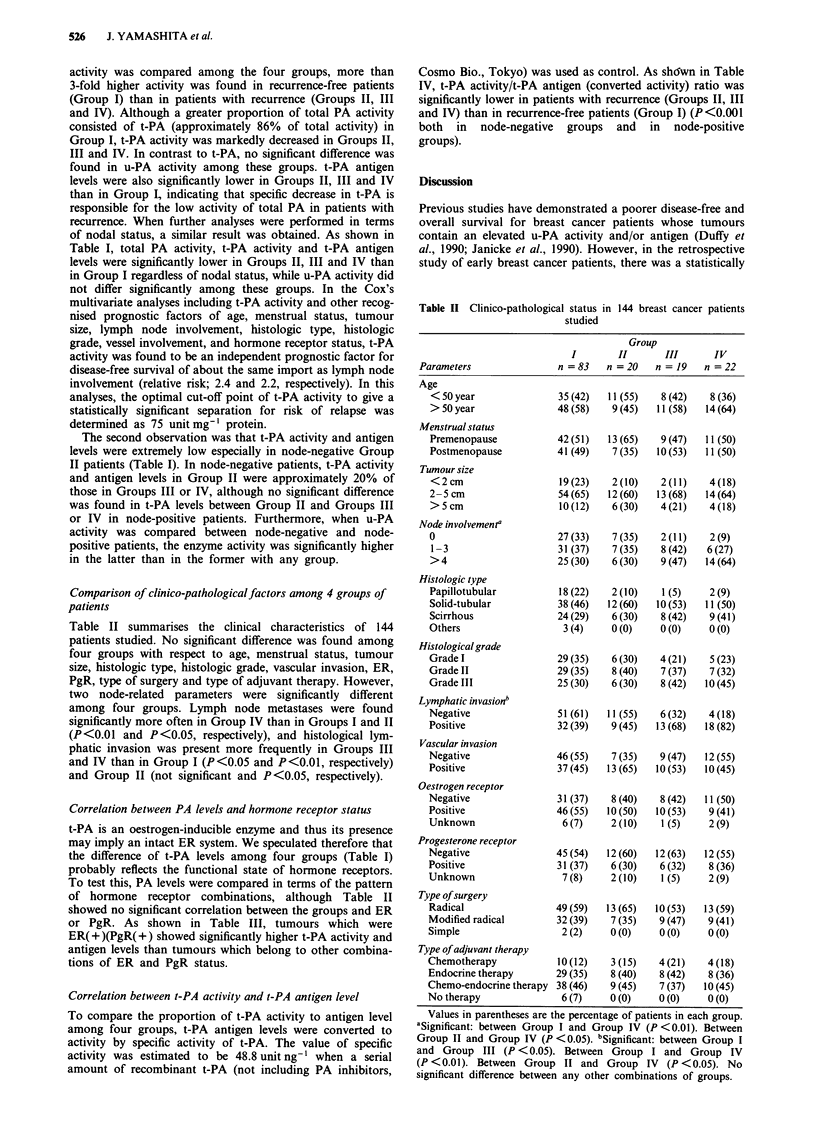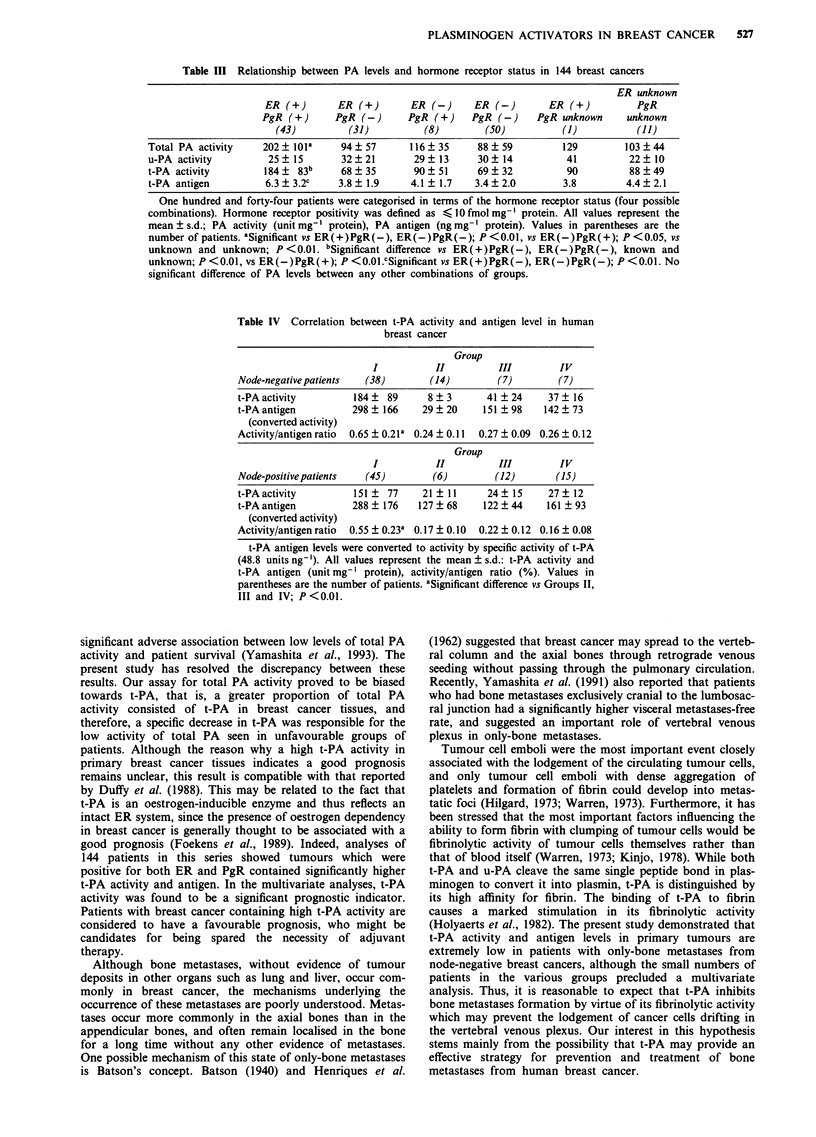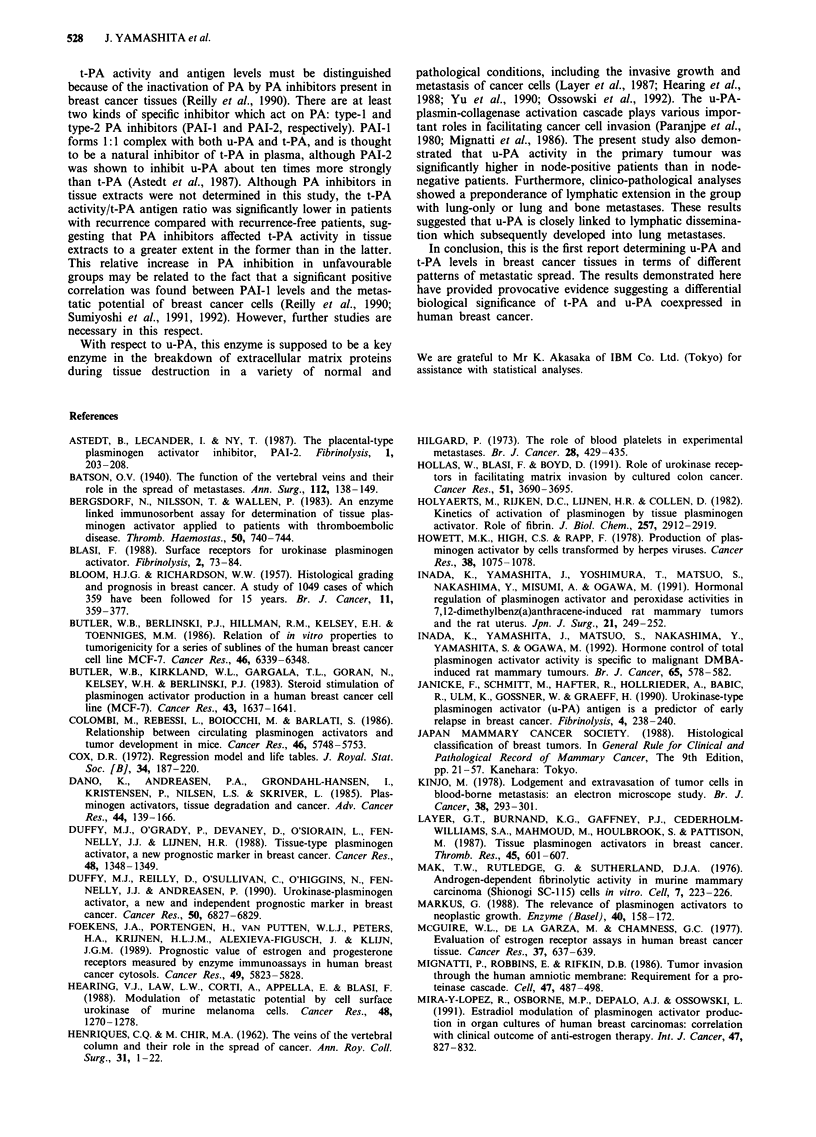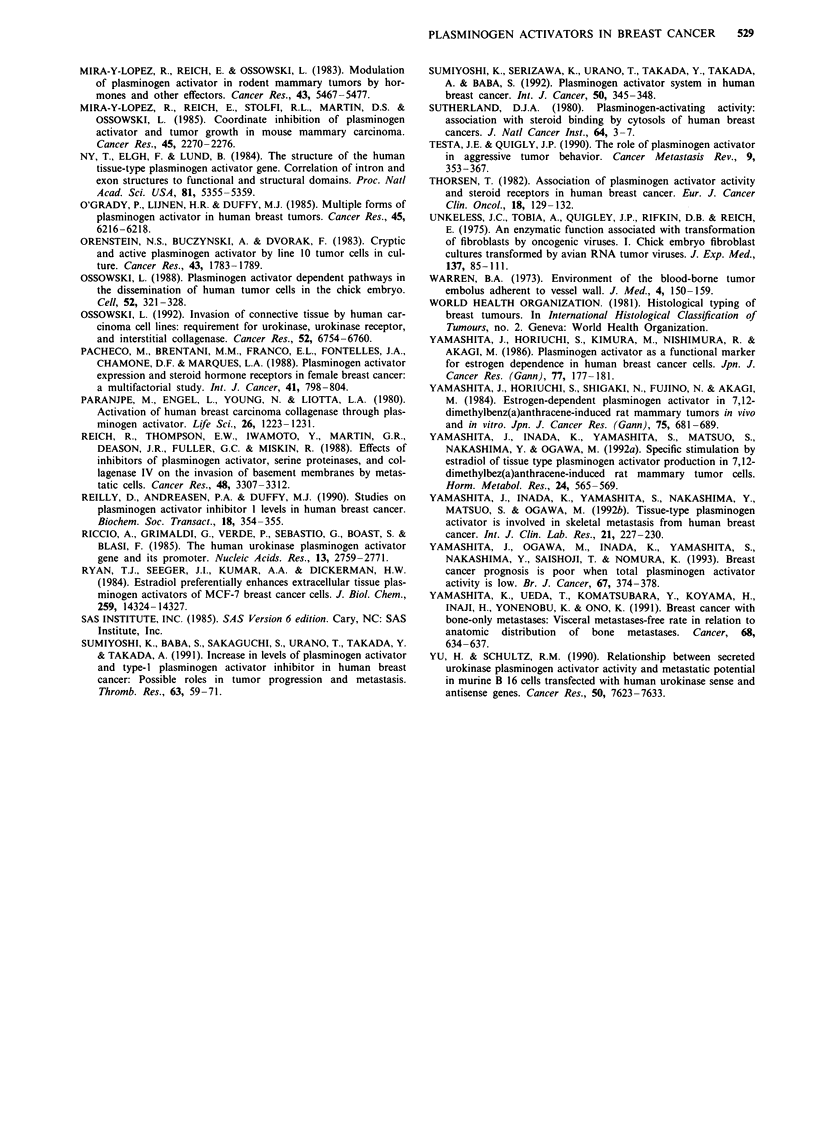# Differential biological significance of tissue-type and urokinase-type plasminogen activator in human breast cancer.

**DOI:** 10.1038/bjc.1993.380

**Published:** 1993-09

**Authors:** J. Yamashita, M. Ogawa, S. Yamashita, Y. Nakashima, T. Saishoji, K. Nomura, K. Inada, I. Kawano

**Affiliations:** Department of Surgery II, Kumamoto University Medical School, Japan.

## Abstract

Plasminogen activator (PA) is a serine protease existing in two forms known as tissue-type (t-PA) and urokinase-type (u-PA). To examine whether PA is related to the postoperative clinical course of human breast cancer, total PA activity, t-PA activity, u-PA activity, and immunoreactive t-PA were determined in tissue extracts from 144 breast cancer specimens. The patients were initially divided into four groups according to the postoperative clinical course: Group I (83 patients who are disease-free), Group II (20 patients whose first metastases were found only in bone), Group III (19 patients whose first metastases were found in both bone and lung), and Group IV (22 patients whose first metastases were found only in lung). Total PA activity was significantly lower in Groups, II, III and IV than in Group I. Both t-PA activity and t-PA antigen levels were also significantly lower in Groups II, III and IV than in Group I, while no significant difference was found in u-PA activity among these groups, indicating that low activity of total PA in Groups II, III and IV was due to a decrease in t-PA but not in u-PA. In the multivariate analyses, t-PA activity was found to be an independent prognostic factor for relapse-free survival. When four groups of patients were further analysed in terms of nodal status, both t-PA activity and antigen levels were markedly decreased in the node-negative Group II compared with the node-negative Groups III and IV or with the node-positive Groups II, III and IV. Of additional interest, u-PA activity was significantly higher in node-positive patients than in node-negative patients with any group. The clinico-pathologic analyses of the patients in this series showed that node involvement and lymphatic invasion were more frequently positive in Groups III and IV than in Groups I and II. When 144 breast cancers were categorised in terms of combinations of oestrogen receptor (ER) and progesterone receptor (PgR) status, breast cancers which were positive for both receptors were found to contain the highest t-PA activity and antigen. This study provides provocative evidence suggesting a possible differential significance of t-PA and u-PA expression in human breast cancer.


Br. J. Cancer (1993), 68, 524–529 © Macmillan Press Ltd., 1993

### Differential biological significance of tissue-type and urokinase-type plasminogen activator in human breast cancer

J. Yamashita, M. Ogawa, S. Yamashita, Y. Nakashima, T. Saishoji, K. Nomura, K. Inada & I. Kawano

*Department of Surgery II, Kumamoto University Medical School, Honjo 1-1-1, Kumamoto 860, Japan.*

**Summary** Plasminogen activator (PA) is a serine protease existing in two forms known as tissue-type (t-PA) and urokinase-type (u-PA). To examine whether PA is related to the postoperative clinical course of human breast cancer, total PA activity, t-PA activity, u-PA activity, and immunoreactive t-PA were determined in tissue extracts from 144 breast cancer specimens. The patients were initially divided into four groups according to the postoperative clinical course: Group I (83 patients who are disease-free), Group II (20 patients whose first metastases were found only in bone), Group III (19 patients whose first metastases were found in both bone and lung), and Group IV (22 patients whose first metastases were found only in lung). Total PA activity was significantly lower in Groups, II, III and IV than in Group I. Both t-PA activity and t-PA antigen levels were also significantly lower in Groups II, III and IV than in Group I, while no significant difference was found in u-PA activity among these groups, indicating that low activity of total PA in Groups II, III and IV was due to a decrease in t-PA but not in u-PA. In the multivariate analyses, t-PA activity was found to be an independent prognostic factor for relapse-free survival. When four groups of patients were further analysed in terms of nodal status, both t-PA activity and antigen levels were markedly decreased in the node-negative Group II compared with the node-negative Groups III and IV or with the node-positive Groups II, III and IV. Of additional interest, u-PA activity was significantly higher in node-positive patients than in node-negative patients with any group. The clinico-pathologic analyses of the patients in this series showed that node involvement and lymphatic invasion were more frequently positive in Groups III and IV than in Groups I and II. When 144 breast cancers were categorised in terms of combinations of oestrogen receptor (ER) and progesterone receptor (PgR) status, breast cancers which were positive for both receptors were found to contain the highest t-PA activity and antigen. This study provides provocative evidence suggesting a possible differential significance of t-PA and u-PA expression in human breast cancer.

Many transformed or malignant tumour cells are known to produce plasminogen activator (PA), a serine-type protease converting plasminogen to plasmin (Unkeless *et al.*, 1975; Howett *et al.*, 1978; Orenstein *et al.*, 1983). It is widely accepted that PA is intimately involved in the metastatic spread of tumour cells (Dano *et al.*, 1985; Colombi *et al.*, 1986; Mignatti *et al.*, 1986; Reich *et al.*, 1988; Ossowski *et al.*, 1988; Markus *et al.*, 1988; Yu *et al.*, 1990; Testa *et al.*, 1990; Hollas *et al.*, 1991). There are two main forms of PA: urokinase type (u-PA) and tissue-type (t-PA). While both catalyse cleavage of the peptide bond between Arg-Val in plasminogen, thus converting the proenzyme to plasmin, they differ in many aspects such as their molecular weight, immunological reactivity and amino acid sequence (Ny *et al.*, 1984; Riccio *et al.*, 1985; Blasi *et al.*, 1988).

PA expressed in breast cancer tissue has attracted attention as (i) it is induced by oestrogen in breast cancer cells in culture (Butler *et al.*, 1983; Ryan *et al.*, 1984; Yamashita *et al.*, 1984, 1992a; Inada *et al.*, 1991, 1992; Mira-y-Lopez *et al.*, 1991), (ii) it correlates with oestrogen receptor (ER) and/or progesterone receptor (PgR) content in human breast cancer (Sutherland, 1980; Thorsen, 1982; Yamashita *et al.*, 1986; Pacheco *et al.*, 1988), and (iii) there are analogous effects of hormones, including oestrogen, on breast cancer growth and PA expression (Mak *et al.*, 1976; Mira-y-Lopez *et al.*, 1983, 1985; Butler *et al.*, 1986). In a multivariate analysis we recently showed a significant association between low levels of total PA activity and poor prognosis of breast cancer patients and suggested the value of total PA assays as a prognostic indicator for disease-free and overall survival (Yamashita *et al.*, 1993). This was unexpected in the light of previous publications that showed a poorer disease-free and overall survival for breast cancer patients whose tumours contain an elevated u-PA activity and/or antigen (Duffy *et al.*, 1990; Janicke *et al.*, 1990). In this study, we therefore evaluated the t-PA and u-PA components of total activity, in the hope that this might resolve the apparent discrepancy between our report and those of others.

We have previously measured the concentration of t-PA in tissue extracts from 27 human breast cancers and presented preliminary evidence that patients with only-bone metastases had significantly less t-PA antigen level in their primary breast tumours than those with metastases to other organs (Yamashita *et al.*, 1992*b*). We have now extended this study and determined not only t-PA antigen level but also t-PA and u-PA activities in primary breast cancer tissues. These enzyme activities are compared with the postoperative clinical course of the patients. We report here that (i) a greater proportion of total PA activity is composed of t-PA in primary breast cancer tissues, and a decrease in t-PA, but not u-PA, is responsible for the low activity of total PA in patients with poor prognosis, (ii) t-PA activity and antigen levels are extremely low in patients with only-bone metastases from node-negative breast cancers, (iii) it is u-PA rather than t-PA that is involved in lymphatic dissemination which might subsequently develop into lung metastases. A possible differential significance of t-PA and u-PA expression in human breast cancer is discussed.

### Patients and methods

### *Patients*

This retrospective study was based on the records of 144 breast cancer patients who underwent curative mastectomy in the Department of Surgery II, Kumamoto University Hospital, during the 6 year period from 1981 to 1986. The median follow-up period for patients was 7.7 years (range, 6.6–11.2 years). These patients were divided into four groups according to the postoperative clinical course: Group I (83 patients who remained free of distant metastases), Group II (20 patients whose first metastases were found only in bone and remained confined in bone within 12 months), Group III (19 patients whose first metastases were found in both bone

Correspondence: M. Ogawa, Department of Surgery II, Kumamoto University Medical School, Honjo 1-1-1, Kumamoto 860, Japan.
Received 25 February 1993; and in revised form 11 May 1993.

and lung simultaneously, or those whose first metastases were found in bone or lung and subsequently detected in the other site within 12 months), and Group IV (22 patients whose first metastases were found only in lung and remained confined in lung within 12 months. Group III included 3 patients who had also liver metastases. The median follow-up for patients in each group was: Group I, 9.1 years; Group II, 7.9 years; Group III, 7.2 years; and Group IV, 6.6 years.

The routine follow-up of these patients after surgery consisted of clinical evaluation every 3 months in the first year and every 6 months thereafter. Disease recurrence was documented by physical examination and radiological and laboratory tests. At the time that bone metastases were first documented, a bone scanning and bone roentgenograph were used for the detection of the metastatic bone lesions. Also, at the time that lung metastases were first documented, chest roentgenograph and computed tomographic (CT) scanning of the lung were used. At the time that the first recurrence was discovered, a bone scanning, radiography or CT scanning of the lung, CT or ultrasonic scanning of the liver, CT scanning of the brain, and if necessary lymph node or skin biopsy were performed to exclude metastases in other sites.

The clinico-pathologic parameters reviewed in this study were age, menstrual status, tumour size, node involvement, histologic type, histologic grade, lymphatic invasion, vascular invasion, ER, PgR, type of surgery and type of adjuvant therapy. Tumour size was recorded at the greatest diameter of the tumour. The extent of lymph node metastases was categorised into one of three groups: 0, 1 to 3 and 4+. When histopathological typing was performed in 144 breast cancers according to the WHO classification (1981), all tumours in our series were classified into the same category, i.e., invasive ductal carcinoma. Therefore, each tumour was further analysed according to the classification of the Japanese Breast Cancer Society (1988) and was graded in parallel according to the criteria described by Bloom and Richardson (1957). ER and PgR were determined by the dextran-coated charcoal method as described previously (McGuire *et al.*, 1977). Tumour specimens were considered hormone receptor-positive if they contained at least 10 fmol specific binding sites $mg^{-1}$ protein.

### *Assay for PA*

The analyses in this study focused on whether or not the PA levels in the primary tumours differed among four groups. Samples for enzyme assay were prepared using 50 mM Tris-HCl buffer (pH 7.4) containing 0.25% Triton X-100, as described previously (Yamashita *et al.*, 1984). Total PA activity was determined as described previously (Yamashita *et al.*, 1984) in a coupled assay using S-2251 (H-D-Val-Leu-Lys-pNA, Kabi, Stockholm) as a substrate for plasmin. t-PA and u-PA activities were assayed according to the method of O'Grady *et al.* (1985) except that monoclonal antibodies to t-PA and u-PA (Cosmo Bio., Tokyo) were employed. Briefly, to measure the t-PA and u-PA activities, total PA activity was determined in the presence and absence of quenching antibodies against t-PA and u-PA. The activity in the presence of anti-t-PA antibody and anti-u-PA antibody was regarded as the u-PA and t-PA activity, respectively. In most cases, the proportion of activity quenched by anti-t-PA and anti-u-PA antibodies always added up to approximately 100%, indicating that t-PA and u-PA were the only forms of total PA present in the tissue extracts. The t-PA antigen level was determined with an enzyme-linked immunosorbent assay (ELISA) described by Bergsdorf *et al.* (1983).

### *Statistical analyses*

Non-parametric statistics were used throughout. For comparing differences between two different groups, the Mann-Whitney *U*-test was used. For database management and descriptive statistics, the SAS program (1985) was used. Life-table analysis and Cox analysis (1972) were performed using the BMDP statistical package program for the computer (IBM 4381, IBM, New York).

### Results

### *Correlation between PA levels and metastasis status*

Table I shows the correlation between PA levels and post-operative clinical course of 144 patients. When total PA

**Table I** Comparison of PA levels among four groups of patients

| | *Group* | | | |
|---|---|---|---|---|
| *Total patients* | *I (83)* | *II (20)* | *III (19)* | *IV (22)* |
| Total PA activity | 184 ± 95[a] | 39 ± 26 | 50 ± 27 | 54 ± 27 |
| u-PA activity | 28 ± 17 | 31 ± 11 | 24 ± 10 | 31 ± 16 |
| t-PA activity | 166 ± 82[a] | 12 ± 5[b] | 30 ± 19 | 30 ± 14 |
| t-PA antigen | 6.0 ± 3.5[a] | 1.2 ± 0.7[b] | 2.7 ± 1.3 | 3.2 ± 1.8 |
| | *Group* | | | |
| *Node-negative patients* | *I (38)* | *II (14)* | *III (7)* | *IV (7)* |
| Total PA activity | 191 ± 103[a] | 33 ± 24 | 58 ± 34 | 51 ± 25 |
| u-PA activity | 22 ± 13[e] | 27 ± 11[f] | 18 ± 9[f] | 20 ± 11[f] |
| t-PA activity | 184 ± 89[a] | 8 ± 3[b,g] | 41 ± 24 | 37 ± 16 |
| t-PA antigen | 6.1 ± 3.4[c] | 0.6 ± 0.4[b] | 3.1 ± 2.0 | 2.9 ± 1.5 |
| | *Group* | | | |
| *Node-positive patients* | *I (45)* | *II (6)* | *III (12)* | *IV (15)* |
| Total PA activity | 178 ± 89[a] | 54 ± 32 | 46 ± 19 | 55 ± 27 |
| u-PA activity | 33 ± 21 | 39 ± 10 | 28 ± 10 | 36 ± 17 |
| t-PA activity | 151 ± 77[a] | 21 ± 11 | 24 ± 15 | 27 ± 12 |
| t-PA antigen | 5.9 ± 3.6[d] | 2.6 ± 1.4 | 2.5 ± 0.9 | 3.3 ± 1.9 |

All values represent the mean ± s.d.; PA activity (unit $mg^{-1}$ protein), PA antigen (ng $mg^{-1}$ protein). Values in parentheses are the number of patients. [a]Significant *vs* Groups II, III and IV; $P<0.01$. [b]Significant *vs* Groups I, III and IV; $P<0.01$. [c]Significant *vs* Group II; $P<0.01$, *vs* Group III; $P<0.05$, *vs* Group IV; $P<0.05$. [d]Significant *vs* Group II; $P<0.05$, *vs* Group III; $P<0.01$, *vs* Group IV; $P<0.01$. [e]Significant *vs* u-PA activity of node-positive Group I; $P<0.01$. [f]Significant *vs* u-PA activity of respective node-positive groups; $P<0.05$. [g]Significant *vs* t-PA activity of node-positive Group II; $P<0.01$. No significant difference of PA levels between any other combinations of groups.

activity was compared among the four groups, more than 3-fold higher activity was found in recurrence-free patients (Group I) than in patients with recurrence (Groups II, III and IV). Although a greater proportion of total PA activity consisted of t-PA (approximately 86% of total activity) in Group I, t-PA activity was markedly decreased in Groups II, III and IV. In contrast to t-PA, no significant difference was found in u-PA activity among these groups. t-PA antigen levels were also significantly lower in Groups II, III and IV than in Group I, indicating that specific decrease in t-PA is responsible for the low activity of total PA in patients with recurrence. When further analyses were performed in terms of nodal status, a similar result was obtained. As shown in Table I, total PA activity, t-PA activity and t-PA antigen levels were significantly lower in Groups II, III and IV than in Group I regardless of nodal status, while u-PA activity did not differ significantly among these groups. In the Cox's multivariate analyses including t-PA activity and other recognised prognostic factors of age, menstrual status, tumour size, lymph node involvement, histologic type, histologic grade, vessel involvement, and hormone receptor status, t-PA activity was found to be an independent prognostic factor for disease-free survival of about the same import as lymph node involvement (relative risk; 2.4 and 2.2, respectively). In this analyses, the optimal cut-off point of t-PA activity to give a statistically significant separation for risk of relapse was determined as 75 unit $mg^{-1}$ protein.

The second observation was that t-PA activity and antigen levels were extremely low especially in node-negative Group II patients (Table I). In node-negative patients, t-PA activity and antigen levels in Group II were approximately 20% of those in Groups III or IV, although no significant difference was found in t-PA levels between Group II and Groups III or IV in node-positive patients. Furthermore, when u-PA activity was compared between node-negative and node-positive patients, the enzyme activity was significantly higher in the latter than in the former with any group.

*Comparison of clinico-pathological factors among 4 groups of patients*

Table II summarises the clinical characteristics of 144 patients studied. No significant difference was found among four groups with respect to age, menstrual status, tumour size, histologic type, histologic grade, vascular invasion, ER, PgR, type of surgery and type of adjuvant therapy. However, two node-related parameters were significantly different among four groups. Lymph node metastases were found significantly more often in Group IV than in Groups I and II ($P<0.01$ and $P<0.05$, respectively), and histological lymphatic invasion was present more frequently in Groups III and IV than in Group I ($P<0.05$ and $P<0.01$, respectively) and Group II (not significant and $P<0.05$, respectively).

*Correlation between PA levels and hormone receptor status*

t-PA is an oestrogen-inducible enzyme and thus its presence may imply an intact ER system. We speculated therefore that the difference of t-PA levels among four groups (Table I) probably reflects the functional state of hormone receptors. To test this, PA levels were compared in terms of the pattern of hormone receptor combinations, although Table II showed no significant correlation between the groups and ER or PgR. As shown in Table III, tumours which were ER(+)(PgR(+) showed significantly higher t-PA activity and antigen levels than tumours which belong to other combinations of ER and PgR status.

*Correlation between t-PA activity and t-PA antigen level*

To compare the proportion of t-PA activity to antigen level among four groups, t-PA antigen levels were converted to activity by specific activity of t-PA. The value of specific activity was estimated to be 48.8 unit $ng^{-1}$ when a serial amount of recombinant t-PA (not including PA inhibitors, Cosmo Bio., Tokyo) was used as control. As shown in Table IV, t-PA activity/t-PA antigen (converted activity) ratio was significantly lower in patients with recurrence (Groups II, III and IV) than in recurrence-free patients (Group I) ($P<0.001$ both in node-negative groups and in node-positive groups).

### Discussion

Previous studies have demonstrated a poorer disease-free and overall survival for breast cancer patients whose tumours contain an elevated u-PA activity and/or antigen (Duffy *et al.*, 1990; Janicke *et al.*, 1990). However, in the retrospective study of early breast cancer patients, there was a statistically

**Table II** Clinico-pathological status in 144 breast cancer patients studied

| *Parameters* | *Group I* n = 83 | *II* n = 20 | *III* n = 19 | *IV* n = 22 |
|---|---|---|---|---|
| Age | | | | |
| < 50 year | 35 (42) | 11 (55) | 8 (42) | 8 (36) |
| > 50 year | 48 (58) | 9 (45) | 11 (58) | 14 (64) |
| *Menstrual status* | | | | |
| Premenopause | 42 (51) | 13 (65) | 9 (47) | 11 (50) |
| Postmenopause | 41 (49) | 7 (35) | 10 (53) | 11 (50) |
| *Tumour size* | | | | |
| < 2 cm | 19 (23) | 2 (10) | 2 (11) | 4 (18) |
| 2–5 cm | 54 (65) | 12 (60) | 13 (68) | 14 (64) |
| > 5 cm | 10 (12) | 6 (30) | 4 (21) | 4 (18) |
| *Node involvement*[a] | | | | |
| 0 | 27 (33) | 7 (35) | 2 (11) | 2 (9) |
| 1–3 | 31 (37) | 7 (35) | 8 (42) | 6 (27) |
| > 4 | 25 (30) | 6 (30) | 9 (47) | 14 (64) |
| *Histologic type* | | | | |
| Papillotubular | 18 (22) | 2 (10) | 1 (5) | 2 (9) |
| Solid-tubular | 38 (46) | 12 (60) | 10 (53) | 11 (50) |
| Scirrhous | 24 (29) | 6 (30) | 8 (42) | 9 (41) |
| Others | 3 (4) | 0 (0) | 0 (0) | 0 (0) |
| *Histological grade* | | | | |
| Grade I | 29 (35) | 6 (30) | 4 (21) | 5 (23) |
| Grade II | 29 (35) | 8 (40) | 7 (37) | 7 (32) |
| Grade III | 25 (30) | 6 (30) | 8 (42) | 10 (45) |
| *Lymphatic invasion*[b] | | | | |
| Negative | 51 (61) | 11 (55) | 6 (32) | 4 (18) |
| Positive | 32 (39) | 9 (45) | 13 (68) | 18 (82) |
| *Vascular invasion* | | | | |
| Negative | 46 (55) | 7 (35) | 9 (47) | 12 (55) |
| Positive | 37 (45) | 13 (65) | 10 (53) | 10 (45) |
| *Oestrogen receptor* | | | | |
| Negative | 31 (37) | 8 (40) | 8 (42) | 11 (50) |
| Positive | 46 (55) | 10 (50) | 10 (53) | 9 (41) |
| Unknown | 6 (7) | 2 (10) | 1 (5) | 2 (9) |
| *Progesterone receptor* | | | | |
| Negative | 45 (54) | 12 (60) | 12 (63) | 12 (55) |
| Positive | 31 (37) | 6 (30) | 6 (32) | 8 (36) |
| Unknown | 7 (8) | 2 (10) | 1 (5) | 2 (9) |
| *Type of surgery* | | | | |
| Radical | 49 (59) | 13 (65) | 10 (53) | 13 (59) |
| Modified radical | 32 (39) | 7 (35) | 9 (47) | 9 (41) |
| Simple | 2 (2) | 0 (0) | 0 (0) | 0 (0) |
| *Type of adjuvant therapy* | | | | |
| Chemotherapy | 10 (12) | 3 (15) | 4 (21) | 4 (18) |
| Endocrine therapy | 29 (35) | 8 (40) | 8 (42) | 8 (36) |
| Chemo-endocrine therapy | 38 (46) | 9 (45) | 7 (37) | 10 (45) |
| No therapy | 6 (7) | 0 (0) | 0 (0) | 0 (0) |

Values in parentheses are the percentage of patients in each group. [a]Significant: between Group I and Group IV ($P<0.01$). Between Group II and Group IV ($P<0.05$). [b]Significant: between Group I and Group III ($P<0.05$). Between Group I and Group IV ($P<0.01$). Between Group II and Group IV ($P<0.05$). No significant difference between any other combinations of groups.

**Table III** Relationship between PA levels and hormone receptor status in 144 breast cancers

| | *ER (+) PgR (+) (43)* | *ER (+) PgR (−) (31)* | *ER (−) PgR (+) (8)* | *ER (−) PgR (−) (50)* | *ER (+) PgR unknown (1)* | *ER unknown PgR unknown (11)* |
|---|---|---|---|---|---|---|
| Total PA activity | 202 ± 101[a] | 94 ± 57 | 116 ± 35 | 88 ± 59 | 129 | 103 ± 44 |
| u-PA activity | 25 ± 15 | 32 ± 21 | 29 ± 13 | 30 ± 14 | 41 | 22 ± 10 |
| t-PA activity | 184 ± 83[b] | 68 ± 35 | 90 ± 51 | 69 ± 32 | 90 | 88 ± 49 |
| t-PA antigen | 6.3 ± 3.2[c] | 3.8 ± 1.9 | 4.1 ± 1.7 | 3.4 ± 2.0 | 3.8 | 4.4 ± 2.1 |

One hundred and forty-four patients were categorised in terms of the hormone receptor status (four possible combinations). Hormone receptor positivity was defined as $\leqslant 10$ fmol mg$^{-1}$ protein. All values represent the mean ± s.d.; PA activity (unit mg$^{-1}$ protein), PA antigen (ng mg$^{-1}$ protein). Values in parentheses are the number of patients. [a]Significant *vs* ER(+)PgR(−), ER(−)PgR(−); $P<0.01$, *vs* ER(−)PgR(+); $P<0.05$, *vs* unknown and unknown; $P<0.01$. [b]Significant difference *vs* ER(+)PgR(−), ER(−)PgR(−), known and unknown; $P<0.01$, vs ER(−)PgR(+); $P<0.01$.[c]Significant *vs* ER(+)PgR(−), ER(−)PgR(−); $P<0.01$. No significant difference of PA levels between any other combinations of groups.

**Table IV** Correlation between t-PA activity and antigen level in human breast cancer

| | *Group* | | | |
|---|---|---|---|---|
| *Node-negative patients* | *I (38)* | *II (14)* | *III (7)* | *IV (7)* |
| t-PA activity | 184 ± 89 | 8 ± 3 | 41 ± 24 | 37 ± 16 |
| t-PA antigen (converted activity) | 298 ± 166 | 29 ± 20 | 151 ± 98 | 142 ± 73 |
| Activity/antigen ratio | 0.65 ± 0.21[a] | 0.24 ± 0.11 | 0.27 ± 0.09 | 0.26 ± 0.12 |
| | *Group* | | | |
| *Node-positive patients* | *I (45)* | *II (6)* | *III (12)* | *IV (15)* |
| t-PA activity | 151 ± 77 | 21 ± 11 | 24 ± 15 | 27 ± 12 |
| t-PA antigen (converted activity) | 288 ± 176 | 127 ± 68 | 122 ± 44 | 161 ± 93 |
| Activity/antigen ratio | 0.55 ± 0.23[a] | 0.17 ± 0.10 | 0.22 ± 0.12 | 0.16 ± 0.08 |

t-PA antigen levels were converted to activity by specific activity of t-PA (48.8 units ng$^{-1}$). All values represent the mean ± s.d.: t-PA activity and t-PA antigen (unit mg$^{-1}$ protein), activity/antigen ratio (%). Values in parentheses are the number of patients. [a]Significant difference *vs* Groups II, III and IV; $P<0.01$.

significant adverse association between low levels of total PA activity and patient survival (Yamashita *et al.*, 1993). The present study has resolved the discrepancy between these results. Our assay for total PA activity proved to be biased towards t-PA, that is, a greater proportion of total PA activity consisted of t-PA in breast cancer tissues, and therefore, a specific decrease in t-PA was responsible for the low activity of total PA seen in unfavourable groups of patients. Although the reason why a high t-PA activity in primary breast cancer tissues indicates a good prognosis remains unclear, this result is compatible with that reported by Duffy *et al.* (1988). This may be related to the fact that t-PA is an oestrogen-inducible enzyme and thus reflects an intact ER system, since the presence of oestrogen dependency in breast cancer is generally thought to be associated with a good prognosis (Foekens *et al.*, 1989). Indeed, analyses of 144 patients in this series showed tumours which were positive for both ER and PgR contained significantly higher t-PA activity and antigen. In the multivariate analyses, t-PA activity was found to be a significant prognostic indicator. Patients with breast cancer containing high t-PA activity are considered to have a favourable prognosis, who might be candidates for being spared the necessity of adjuvant therapy.

Although bone metastases, without evidence of tumour deposits in other organs such as lung and liver, occur commonly in breast cancer, the mechanisms underlying the occurrence of these metastases are poorly understood. Metastases occur more commonly in the axial bones than in the appendicular bones, and often remain localised in the bone for a long time without any other evidence of metastases. One possible mechanism of this state of only-bone metastases is Batson's concept. Batson (1940) and Henriques *et al.* (1962) suggested that breast cancer may spread to the vertebral column and the axial bones through retrograde venous seeding without passing through the pulmonary circulation. Recently, Yamashita *et al.* (1991) also reported that patients who had bone metastases exclusively cranial to the lumbosacral junction had a significantly higher visceral metastases-free rate, and suggested an important role of vertebral venous plexus in only-bone metastases.

Tumour cell emboli were the most important event closely associated with the lodgement of the circulating tumour cells, and only tumour cell emboli with dense aggregation of platelets and formation of fibrin could develop into metastatic foci (Hilgard, 1973; Warren, 1973). Furthermore, it has been stressed that the most important factors influencing the ability to form fibrin with clumping of tumour cells would be fibrinolytic activity of tumour cells themselves rather than that of blood itself (Warren, 1973; Kinjo, 1978). While both t-PA and u-PA cleave the same single peptide bond in plasminogen to convert it into plasmin, t-PA is distinguished by its high affinity for fibrin. The binding of t-PA to fibrin causes a marked stimulation in its fibrinolytic activity (Holyaerts *et al.*, 1982). The present study demonstrated that t-PA activity and antigen levels in primary tumours are extremely low in patients with only-bone metastases from node-negative breast cancers, although the small numbers of patients in the various groups precluded a multivariate analysis. Thus, it is reasonable to expect that t-PA inhibits bone metastases formation by virtue of its fibrinolytic activity which may prevent the lodgement of cancer cells drifting in the vertebral venous plexus. Our interest in this hypothesis stems mainly from the possibility that t-PA may provide an effective strategy for prevention and treatment of bone metastases from human breast cancer.

t-PA activity and antigen levels must be distinguished because of the inactivation of PA by PA inhibitors present in breast cancer tissues (Reilly et al., 1990). There are at least two kinds of specific inhibitor which act on PA: type-1 and type-2 PA inhibitors (PAI-1 and PAI-2, respectively). PAI-1 forms 1:1 complex with both u-PA and t-PA, and is thought to be a natural inhibitor of t-PA in plasma, although PAI-2 was shown to inhibit u-PA about ten times more strongly than t-PA (Astedt et al., 1987). Although PA inhibitors in tissue extracts were not determined in this study, the t-PA activity/t-PA antigen ratio was significantly lower in patients with recurrence compared with recurrence-free patients, suggesting that PA inhibitors affected t-PA activity in tissue extracts to a greater extent in the former than in the latter. This relative increase in PA inhibition in unfavourable groups may be related to the fact that a significant positive correlation was found between PAI-1 levels and the metastatic potential of breast cancer cells (Reilly et al., 1990; Sumiyoshi et al., 1991, 1992). However, further studies are necessary in this respect.

With respect to u-PA, this enzyme is supposed to be a key enzyme in the breakdown of extracellular matrix proteins during tissue destruction in a variety of normal and pathological conditions, including the invasive growth and metastasis of cancer cells (Layer et al., 1987; Hearing et al., 1988; Yu et al., 1990; Ossowski et al., 1992). The u-PA-plasmin-collagenase activation cascade plays various important roles in facilitating cancer cell invasion (Paranjpe et al., 1980; Mignatti et al., 1986). The present study also demonstrated that u-PA activity in the primary tumour was significantly higher in node-positive patients than in node-negative patients. Furthermore, clinico-pathological analyses showed a preponderance of lymphatic extension in the group with lung-only or lung and bone metastases. These results suggested that u-PA is closely linked to lymphatic dissemination which subsequently developed into lung metastases.

In conclusion, this is the first report determining u-PA and t-PA levels in breast cancer tissues in terms of different patterns of metastatic spread. The results demonstrated here have provided provocative evidence suggesting a differential biological significance of t-PA and u-PA coexpressed in human breast cancer.

We are grateful to Mr K. Akasaka of IBM Co. Ltd. (Tokyo) for assistance with statistical analyses.

### References

ASTEDT, B., LECANDER, I. & NY, T. (1987). The placental-type plasminogen activator inhibitor, PAI-2. *Fibrinolysis*, **1**, 203–208.

BATSON, O.V. (1940). The function of the vertebral veins and their role in the spread of metastases. *Ann. Surg.*, **112**, 138–149.

BERGSDORF, N., NILSSON, T. & WALLEN, P. (1983). An enzyme linked immunosorbent assay for determination of tissue plasminogen activator applied to patients with thromboembolic disease. *Thromb. Haemostas.*, **50**, 740–744.

BLASI, F. (1988). Surface receptors for urokinase plasminogen activator. *Fibrinolysis*, **2**, 73–84.

BLOOM, H.J.G. & RICHARDSON, W.W. (1957). Histological grading and prognosis in breast cancer. A study of 1049 cases of which 359 have been followed for 15 years. *Br. J. Cancer*, **11**, 359–377.

BUTLER, W.B., BERLINSKI, P.J., HILLMAN, R.M., KELSEY, E.H. & TOENNIGES, M.M. (1986). Relation of *in vitro* properties to tumorigenicity for a series of sublines of the human breast cancer cell line MCF-7. *Cancer Res.*, **46**, 6339–6348.

BUTLER, W.B., KIRKLAND, W.L., GARGALA, T.L., GORAN, N., KELSEY, W.H. & BERLINSKI, P.J. (1983). Steroid stimulation of plasminogen activator production in a human breast cancer cell line (MCF-7). *Cancer Res.*, **43**, 1637–1641.

COLOMBI, M., REBESSI, L., BOIOCCHI, M. & BARLATI, S. (1986). Relationship between circulating plasminogen activators and tumor development in mice. *Cancer Res.*, **46**, 5748–5753.

COX, D.R. (1972). Regression model and life tables. *J. Royal. Stat. Soc. [B]*, **34**, 187–220.

DANO, K., ANDREASEN, P.A., GRONDAHL-HANSEN, I., KRISTENSEN, P., NILSEN, L.S. & SKRIVER, L. (1985). Plasminogen activators, tissue degradation and cancer. *Adv. Cancer Res.*, **44**, 139–166.

DUFFY, M.J., O'GRADY, P., DEVANEY, D., O'SIORAIN, L., FENNELLY, J.J. & LIJNEN, H.R. (1988). Tissue-type plasminogen activator, a new prognostic marker in breast cancer. *Cancer Res.*, **48**, 1348–1349.

DUFFY, M.J., REILLY, D., O'SULLIVAN, C., O'HIGGINS, N., FENNELLY, J.J. & ANDREASEN, P. (1990). Urokinase-plasminogen activator, a new and independent prognostic marker in breast cancer. *Cancer Res.*, **50**, 6827–6829.

FOEKENS, J.A., PORTENGEN, H., VAN PUTTEN, W.L.J., PETERS, H.A., KRIJNEN, H.L.J.M., ALEXIEVA-FIGUSCH, J. & KLIJN, J.G.M. (1989). Prognostic value of estrogen and progesterone receptors measured by enzyme immunoassays in human breast cancer cytosols. *Cancer Res.*, **49**, 5823–5828.

HEARING, V.J., LAW, L.W., CORTI, A., APPELLA, E. & BLASI, F. (1988). Modulation of metastatic potential by cell surface urokinase of murine melanoma cells. *Cancer Res.*, **48**, 1270–1278.

HENRIQUES, C.Q. & M. CHIR, M.A. (1962). The veins of the vertebral column and their role in the spread of cancer. *Ann. Roy. Coll. Surg.*, **31**, 1–22.

HILGARD, P. (1973). The role of blood platelets in experimental metastases. *Br. J. Cancer*. **28**, 429–435.

HOLLAS, W., BLASI, F. & BOYD, D. (1991). Role of urokinase receptors in facilitating matrix invasion by cultured colon cancer. *Cancer Res.*, **51**, 3690–3695.

HOLYAERTS, M., RIJKEN, D.C., LIJNEN, H.R. & COLLEN, D. (1982). Kinetics of activation of plasminogen by tissue plasminogen activator. Role of fibrin. *J. Biol. Chem.*, **257**, 2912–2919.

HOWETT, M.K., HIGH, C.S. & RAPP, F. (1978). Production of plasminogen activator by cells transformed by herpes viruses. *Cancer Res.*, **38**, 1075–1078.

INADA, K., YAMASHITA, J., YOSHIMURA, T., MATSUO, S., NAKASHIMA, Y., MISUMI, A. & OGAWA, M. (1991). Hormonal regulation of plasminogen activator and peroxidase activities in 7,12-dimethylbenz(a)anthracene-induced rat mammary tumors and the rat uterus. *Jpn. J. Surg.*, **21**, 249–252.

INADA, K., YAMASHITA, J., MATSUO, S., NAKASHIMA, Y., YAMASHITA, S. & OGAWA, M. (1992). Hormone control of total plasminogen activator activity is specific to malignant DMBA-induced rat mammary tumours. *Br. J. Cancer*, **65**, 578–582.

JANICKE, F., SCHMITT, M., HAFTER, R., HOLLRIEDER, A., BABIC, R., ULM, K., GOSSNER, W. & GRAEFF, H. (1990). Urokinase-type plasminogen activator (u-PA) antigen is a predictor of early relapse in breast cancer. *Fibrinolysis*, **4**, 238–240.

JAPAN MAMMARY CANCER SOCIETY. (1988). Histological classification of breast tumors. In *General Rule for Clinical and Pathological Record of Mammary Cancer*, The 9th Edition, pp. 21–57. Kanehara: Tokyo.

KINJO, M. (1978). Lodgement and extravasation of tumor cells in blood-borne metastasis: an electron microscope study. *Br. J. Cancer*, **38**, 293–301.

LAYER, G.T., BURNAND, K.G., GAFFNEY, P.J., CEDERHOLM-WILLIAMS, S.A., MAHMOUD, M., HOULBROOK, S. & PATTISON, M. (1987). Tissue plasminogen activators in breast cancer. *Thromb. Res.*, **45**, 601–607.

MAK, T.W., RUTLEDGE, G. & SUTHERLAND, D.J.A. (1976). Androgen-dependent fibrinolytic activity in murine mammary carcinoma (Shionogi SC-115) cells *in vitro*. *Cell*, **7**, 223–226.

MARKUS, G. (1988). The relevance of plasminogen activators to neoplastic growth. *Enzyme (Basel)*, **40**, 158–172.

MCGUIRE, W.L., DE LA GARZA, M. & CHAMNESS, G.C. (1977). Evaluation of estrogen receptor assays in human breast cancer tissue. *Cancer Res.*, **37**, 637–639.

MIGNATTI, P., ROBBINS, E. & RIFKIN, D.B. (1986). Tumor invasion through the human amniotic membrane: Requirement for a proteinase cascade. *Cell*, **47**, 487–498.

MIRA-Y-LOPEZ, R., OSBORNE, M.P., DEPALO, A.J. & OSSOWSKI, L. (1991). Estradiol modulation of plasminogen activator production in organ cultures of human breast carcinomas: correlation with clinical outcome of anti-estrogen therapy. *Int. J. Cancer*, **47**, 827–832.

MIRA-Y-LOPEZ, R., REICH, E. & OSSOWSKI, L. (1983). Modulation of plasminogen activator in rodent mammary tumors by hormones and other effectors. *Cancer Res.*, **43**, 5467–5477.

MIRA-Y-LOPEZ, R., REICH, E., STOLFI, R.L., MARTIN, D.S. & OSSOWSKI, L. (1985). Coordinate inhibition of plasminogen activator and tumor growth in mouse mammary carcinoma. *Cancer Res.*, **45**, 2270–2276.

NY, T., ELGH, F. & LUND, B. (1984). The structure of the human tissue-type plasminogen activator gene. Correlation of intron and exon structures to functional and structural domains. *Proc. Natl Acad. Sci. USA*, **81**, 5355–5359.

O'GRADY, P., LIJNEN, H.R. & DUFFY, M.J. (1985). Multiple forms of plasminogen activator in human breast tumors. *Cancer Res.*, **45**, 6216–6218.

ORENSTEIN, N.S., BUCZYNSKI, A. & DVORAK, F. (1983). Cryptic and active plasminogen activator by line 10 tumor cells in culture. *Cancer Res.*, **43**, 1783–1789.

OSSOWSKI, L. (1988). Plasminogen activator dependent pathways in the dissemination of human tumor cells in the chick embryo. *Cell*, **52**, 321–328.

OSSOWSKI, L. (1992). Invasion of connective tissue by human carcinoma cell lines: requirement for urokinase, urokinase receptor, and interstitial collagenase. *Cancer Res.*, **52**, 6754–6760.

PACHECO, M., BRENTANI, M.M., FRANCO, E.L., FONTELLES, J.A., CHAMONE, D.F. & MARQUES, L.A. (1988). Plasminogen activator expression and steroid hormone receptors in female breast cancer: a multifactorial study. *Int. J. Cancer*, **41**, 798–804.

PARANJPE, M., ENGEL, L., YOUNG, N. & LIOTTA, L.A. (1980). Activation of human breast carcinoma collagenase through plasminogen activator. *Life Sci.*, **26**, 1223–1231.

REICH, R., THOMPSON, E.W., IWAMOTO, Y., MARTIN, G.R., DEASON, J.R., FULLER, G.C. & MISKIN, R. (1988). Effects of inhibitors of plasminogen activator, serine proteinases, and collagenase IV on the invasion of basement membranes by metastatic cells. *Cancer Res.*, **48**, 3307–3312.

REILLY, D., ANDREASEN, P.A. & DUFFY, M.J. (1990). Studies on plasminogen activator inhibitor 1 levels in human breast cancer. *Biochem. Soc. Transact.*, **18**, 354–355.

RICCIO, A., GRIMALDI, G., VERDE, P., SEBASTIO, G., BOAST, S. & BLASI, F. (1985). The human urokinase plasminogen activator gene and its promoter. *Nucleic Acids. Res.*, **13**, 2759–2771.

RYAN, T.J., SEEGER, J.I., KUMAR, A.A. & DICKERMAN, H.W. (1984). Estradiol preferentially enhances extracellular tissue plasminogen activators of MCF-7 breast cancer cells. *J. Biol. Chem.*, **259**, 14324–14327.

SAS INSTITUTE, INC. (1985). *SAS Version 6 edition*. Cary, NC: SAS Institute, Inc.

SUMIYOSHI, K., BABA, S., SAKAGUCHI, S., URANO, T., TAKADA, Y. & TAKADA, A. (1991). Increase in levels of plasminogen activator and type-1 plasminogen activator inhibitor in human breast cancer: Possible roles in tumor progression and metastasis. *Thromb. Res.*, **63**, 59–71.

SUMIYOSHI, K., SERIZAWA, K., URANO, T., TAKADA, Y., TAKADA, A. & BABA, S. (1992). Plasminogen activator system in human breast cancer. *Int. J. Cancer*, **50**, 345–348.

SUTHERLAND, D.J.A. (1980). Plasminogen-activating activity: association with steroid binding by cytosols of human breast cancers. *J. Natl Cancer Inst.*, **64**, 3–7.

TESTA, J.E. & QUIGLY, J.P. (1990). The role of plasminogen activator in aggressive tumor behavior. *Cancer Metastasis Rev.*, **9**, 353–367.

THORSEN, T. (1982). Association of plasminogen activator activity and steroid receptors in human breast cancer. *Eur. J. Cancer Clin. Oncol.*, **18**, 129–132.

UNKELESS, J.C., TOBIA, A., QUIGLEY, J.P., RIFKIN, D.B. & REICH, E. (1975). An enzymatic function associated with transformation of fibroblasts by oncogenic viruses. I. Chick embryo fibroblast cultures transformed by avian RNA tumor viruses. *J. Exp. Med.*, **137**, 85–111.

WARREN, B.A. (1973). Environment of the blood-borne tumor embolus adherent to vessel wall. *J. Med.*, **4**, 150–159.

WORLD HEALTH ORGANIZATION. (1981). Histological typing of breast tumours. In *International Histological Classification of Tumours*, no. 2. Geneva: World Health Organization.

YAMASHITA, J., HORIUCHI, S., KIMURA, M., NISHIMURA, R. & AKAGI, M. (1986). Plasminogen activator as a functional marker for estrogen dependence in human breast cancer cells. *Jpn. J. Cancer Res. (Gann)*, **77**, 177–181.

YAMASHITA, J., HORIUCHI, S., SHIGAKI, N., FUJINO, N. & AKAGI, M. (1984). Estrogen-dependent plasminogen activator in 7,12-dimethylbenz(a)anthracene-induced rat mammary tumors *in vivo* and *in vitro*. *Jpn. J. Cancer Res. (Gann)*, **75**, 681–689.

YAMASHITA, J., INADA, K., YAMASHITA, S., MATSUO, S., NAKASHIMA, Y. & OGAWA, M. (1992*a*). Specific stimulation by estradiol of tissue type plasminogen activator production in 7,12-dimethylbez(a)anthracene-induced rat mammary tumor cells. *Horm. Metabol. Res.*, **24**, 565–569.

YAMASHITA, J., INADA, K., YAMASHITA, S., NAKASHIMA, Y., MATSUO, S. & OGAWA, M. (1992*b*). Tissue-type plasminogen activator is involved in skeletal metastasis from human breast cancer. *Int. J. Clin. Lab. Res.*, **21**, 227–230.

YAMASHITA, J., OGAWA, M., INADA, K., YAMASHITA, S., NAKASHIMA, Y., SAISHOJI, T. & NOMURA, K. (1993). Breast cancer prognosis is poor when total plasminogen activator activity is low. *Br. J. Cancer*, **67**, 374–378.

YAMASHITA, K., UEDA, T., KOMATSUBARA, Y., KOYAMA, H., INAJI, H., YONENOBU, K. & ONO, K. (1991). Breast cancer with bone-only metastases: Visceral metastases-free rate in relation to anatomic distribution of bone metastases. *Cancer*, **68**, 634–637.

YU, H. & SCHULTZ, R.M. (1990). Relationship between secreted urokinase plasminogen activator activity and metastatic potential in murine B 16 cells transfected with human urokinase sense and antisense genes. *Cancer Res.*, **50**, 7623–7633.